# A precision environmental health approach to prevention of human disease

**DOI:** 10.1038/s41467-023-37626-2

**Published:** 2023-04-28

**Authors:** Andrea Baccarelli, Dana C. Dolinoy, Cheryl Lyn Walker

**Affiliations:** 1 Department of Environmental Health Sciences, Columbia University Mailman School of Public Health, New York, NY USA; 2 Department of Environmental Health Sciences, University of Michigan School of Public Health, Ann Arbor, MI USA; 3 Center for Precision Environmental Health, Baylor College of Medicine, Houston, TX USA

**Keywords:** Molecular medicine, Physiology, Risk factors

## Abstract

Human health is determined by the interaction of our environment with the genome, epigenome, and microbiome, which shape the transcriptomic, proteomic, and metabolomic landscape of cells and tissues. Precision environmental health is an emerging field leveraging environmental and system-level (‘omic) data to understand underlying environmental causes of disease, identify biomarkers of exposure and response, and develop new prevention and intervention strategies. In this article we provide real-life illustrations of the utility of precision environmental health approaches, identify current challenges in the field, and outline new opportunities to promote health through a precision environmental health framework.

## Abstract (web summary)

Precision environmental health leverages environmental and system-level data to understand underlying environmental causes of disease, identify biomarkers of exposure, and develop new prevention and intervention strategies. In this Perspective, the authors provide real-life illustrations of the utility of precision environmental health approaches and identify current challenges in the field.

## Introduction

Our health is affected by the world around us—the climate in which we live, the air we breathe, the water we drink, and the food we eat all impact our health^1^. The broadest interpretation of our environment encompasses all aspects of the world that surrounds us, including our built, socioeconomic, chemical, physiological, and psychosocial environments^2,3^. These diverse components influence how all body systems function, constituting major, and often modifiable, determinants of human health^4^.

Environmental exposures contribute to an estimated 70–90% of the burden of human disease^5^. This relationship between the environment and health is the focus of environmental health science (EHS) research, which seeks to understand how the environment influences human health and promotes healthier lives. In the past, EHS research largely focused on defining and mitigating environmental hazards^6^. Modern EHS research also encompasses beneficial exposures such as essential trace elements, beneficial microbiota, and social support systems. Traditionally, EHS research was translated into action through strategies to reduce harmful exposures, for instance, through policy or technological advances. However, these traditional approaches have limited capacity to consider everyone’s unique combination of type, level, and timing of exposures that, combined with their genetic and patho- and physiological states, are likely to produce distinct health outcomes in each of us. Precision environmental health seeks to tackle this challenge by increasing our understanding of exposures over the lifecourse and determinants of individualized response to these exposures. Identification of individuals at greatest risk for, or who are disproportionately impacted by, diseases linked to environmental exposures is key to enabling precise, targeted, and effective prevention and intervention strategies (Box 1).

The concept of precision environmental health is based on an appreciation that the impact environmental exposures have on health differs among individuals and across populations and time^7^, as well as across life stages^8^. Large interindividual differences exist in genomic, epigenomic, and other molecular and lifestyle factors that contribute to health and risk of disease. Specific life stages, such as during embryo and fetal development, early postnatal life, or aging, have been proposed as discrete windows with higher vulnerability^8^. Environmental exposures themselves are unevenly distributed across populations due to geographic, socioeconomic, and other demographic factors^9,10^, with patterns of concern influenced by environmental injustice and inequity. However, achieving the promise of precision environmental health will require new knowledge, computational approaches, and technologies to move into clinical and public health practice (Box 2). All of these will be needed to evolve our understanding of the relationships between environmental exposures and health and translate that understanding into effective, targeted, and precise real-life applications^11–13^.

Box 1 The future of precision environmental health Imagine a future when you could take a simple blood test to determine if you have been exposed to chemicals in the environment that can harm your health. Your healthcare provider would then offer solutions to ameliorate that exposure and/or prevent its adverse health effects. Or imagine a personal monitoring device that can reveal how you are responding to fluctuations in your environmental exposures caused by climate change. While climate change is global, each of us may be impacted differently, for example, by temperature extremes or inhalation exposures from drought-provoked fires, and will have unique responses. Precision environmental health offers the promise to understand and identify these types of individualized responses and provide precision interventions to improve health and prevent disease.

Box 2 The promise of precision environmental health Precision environmental health seeks to promote health and reduce or eliminate the adverse health effects of environmental exposures while maximizing positive health influences^1^. Current clinical and public health approaches to achieve this goal have not yet fully utilized system-level data-omic approaches to characterize environmental exposures and design strategies to mitigate risk at the individual level^9^. These exposures, sometimes referred to as the “exposome” contribute to an individual’s allostatic load, or cumulative burden of chronic stress and life events. A precision environmental health approach leverages genomics, epigenomics, and other ‘omics, as well as knowledge of the many environmental exposures encountered across the lifecourse. Combining these types of omic-level data holds promise for precise, individualized health promotion and prevention of diseases linked to environmental exposures.

### Precision environmental health toolbox

While precision medicine originated primarily from *genomics*^14^, precision environmental health increasingly focuses on other targets. Genetics has a significant impact on health, but the genome remains largely static throughout an individual’s lifetime. In contrast, other substrates, such as the epigenome or microbiome, are both a target and a determinant of response to environmental exposures. Often environmental exposures leave imprints on these substrates, like fingerprints, providing opportunities to capture previous exposures and predict future risks of disease^15^. This has led to an appreciation of the need to capture data from multiple ‘omic layers using an integrated, data-driven approach to precisely assess how environmental exposures impact each individual, identify adverse or beneficial environmental factors, and develop precisely targeted interventions to improve health and prevent disease. These ‘omic layers include:

#### Genomics and epigenomics

The Human Genome Project identified ~3 billion human bases and 20,000 human genes, including ~10 million single nucleotide polymorphisms (SNPs). Genomic technologies, along with mechanistic genome editing platforms such as CRISPR^16^, can now determine which genetic variables are most responsible for an individual’s response to their environment. Precision environmental health research continues to leverage these advances and incorporate genomics into individualized assessments of susceptibility to environmental exposures. For example, genetic susceptibility factors that contribute to disease risk have clear utility as susceptibility biomarkers^17^.

However, the genome is static and non-modifiable, and therefore has little utility for use as a record of previous environmental exposures in non-diseased tissue or as a target for interventions to reduce the risk of environmental disease. In contrast, the epigenome is potentially modifiable and thus has become a focus in precision environmental health for its potential as a reporter and biomarker of past exposures, as well as a target for interventions^18,19^. Literally meaning “above the genome”, the epigenome encompasses heritable modifications, such as DNA and histone modifications and changes in chromatin conformation, that affect gene expression without altering the underlying DNA sequence^20^. Traditional molecular epidemiology has viewed how the environment affects health through the lens of the genome–environment interactions (GxE)^21^, or how an individual’s genetics influences their response to the environment. For example, individual differences in metabolizing enzyme polymorphisms that alter chemicals’ toxicity can, in the case of dietary carcinogens, change their potency to influence the risk of developing cancer^22,23^. Now, the field is moving beyond GxE to study interactions between the environment and other system-level substrates, such as the epigenome. Hundreds of in vitro, animal, and human studies over the past decade have now established the importance of epigenome x environment (ExE) interactions. An example of an ExE interaction is exposure to lead and DNA methylation. Data on lead levels from pregnant mothers and DNA methylation from their infants from the Early-Life Exposure in Mexico to Environmental Toxicants (ELEMENT) project show that environmental lead exposures are associated with infant DNA methylation changes in LINE-1 transposable elements^24^. While ELEMENT data do not link those epigenetic changes to childhood outcomes, a separate study does—childhood obesity data from the Healthy Families Study showed that LINE-1 methylation associates with obesity status during childhood^25^.

#### Transcriptomics and epitranscriptomics

Transcriptomic studies using next-generation sequencing can comprehensively profile protein-coding (mRNAs) as well as non-coding RNAs (e.g., microRNAs, long non-coding RNAs, tRNAs), which play an equally important role in the cell as coding RNAs. Most recently, epitranscriptomics^26^—a study of the regulation and function of post-transcriptional RNA modifications—has emerged as an important link between environmental exposures and disease^27–31^. Studies in various model systems show that environmental stressors can change RNA modifications and reprogram regulatory RNAs^32^. Additional research on the impact of environmental exposures on RNA processing and modifications is needed to understand the mechanisms by which exposures perturb RNA, and to link this biology to environmental causes of human disease. In this context, exosomes with targeted RNA cargos^33^, may also provide new approaches to assessing exposure and response, and as tools for interventions^34,35^. Exosome cargos include non-coding RNAs that are responsive to environmental stressors^36,37^. Exosomes and their contents can be engineered to deliver specific cargoes to target tissues, including sense or antisense RNAs that can reverse deficits or counteract overexpressed RNAs in those tissues^38^.

#### Proteomics

Proteomics is the analysis of many, and potentially all, proteins in a specimen^39^. The development of proteomic biomarkers and the ability to detect early protein-level changes in response to environmental exposures holds the potential for the prevention of environmentally induced adverse health effects^40^. For example, proteomic studies have discovered 16 proteins, many related to cardiovascular disease and function, associated with low-level mercury or lead exposure^41^, and protein biomarkers have been identified for fluoride exposure in children^42^. Shotgun proteomic methods [e.g., gel electrophoresis liquid chromatography–tandem mass spectrometry (GeLC-MS/MS)] can identify proteins that are depleted or increased in response to environmental exposures^43^. Additionally, LC-MS/MS-based methods to characterize posttranslational modifications (PTMs) can also be biomarkers if specific PTMs are significantly associated with exposure or toxicity^44^. However, implementing these technologies requires specialized expertise, and often, expensive instrumentation, and targeted proteomics is much further advanced than our current ability to identify all proteins in a specimen. This is further exacerbated by the importance of proteoforms, which is the specific combination of PTMs on a single protein molecule, which provide a level of resolution and information not yet routinely available.

#### Microbiome

Advances in understanding the role of microorganisms in health and disease brought attention to the microbiome as a mediator of environmental health and as a potential target for interventions^45,46^. Indeed, the microbiome is a key interface between exogenous and endogenous exposures and health. It can be modified by environmental exposures, affect health, and even be used in interventions to prevent environmental disease^47^. Some microbes alter environmental substances and make them more toxic, while others make environmental substances less harmful. The microbiome, because it can be modified by simple interventions, including nutritional interventions or probiotics, holds special promise in identifying new approaches to prevent the harmful and amplifying beneficial effects of environmental exposures, for example, to promote cardiovascular health^50^. The interplay of environmental exposures, the microbiome, and the epithelial barrier has also been proposed to underlie the observed increase in allergic and autoimmune disease^48,49^. Metagenomic analyses are key to unlocking environment x microbiome interactions: ribosomal 16 S sequencing can provide information on changes in bacterial composition in a sample, whereas whole genome shotgun (WGS) sequencing provides genome coverage of all organisms in a sample and greater taxonomic (read: functional) resolution.

Despite the wide availability of metagenomic sequencing, more knowledge is needed about crosstalk between the microbiome and other “omic” layers, for example, the metabolome, epigenome, and microbiome. Short-chain fatty acid (SCFA) metabolites, such as acetate and butyrate, are exclusively derived from microbial fermentation of dietary fiber^50^. SCFAs provide the acyl groups needed for several epigenetic histone modifications and are the acyl donors required for the activity of histone acetyltransferases^51–56^. Thus, SCFA production by the microbiome is required for normal epigenomic programming, and when deficient or perturbed by environmental exposures, has been shown to cause reprogramming of the epigenome in multiple tissues^57,58^ Investigating the microbiome-metabolome interplay is critical to toxicology and environmental health. As an example, Lu et al. showed that C57BL/6 mice treated with arsenic have concurrent changes in both the gut microbiome and urine metabolomics^59^. Gao et al. provided a prototype of human multi-omic studies that included high-resolution untargeted exposome-scale analysis of chemicals and metabolites in plasma and gut microbiome analysis, as well as genomic and transcriptomic analyses^11^. This study demonstrated that both time and location impacted all the omic levels investigated and their highly dynamic interplay^11^.

#### Metabolomics

Metabolic homeostasis is intricately regulated and sensitive to environmental conditions, and metabolites can provide insight into the body’s dynamic response to diet, exercise, pharmaceutical use, and exposure to chemical stressors^60^. Metabolic profiling can, for example, provide signatures for and identify responses to environmental exposures as varied as air pollution, persistent organic pollutants, proximity to industrial operations, metals, perfluorinated substances, plasticizers, and climate-related variables^61–63^. An extensive array of metabolic measurements (e.g., targeted metabolomics panels of citric acid cycle intermediates, amino acids, acylcarnitines, free fatty acids, targeted lipidomics, multiplexed hormone analysis) and untargeted metabolomics are possible. However, not all chemical metabolites are persistent, and metabolic perturbations in response to environmental exposures are often labile, and both can be challenging to capture. However, metabolic analyses are becoming more readily available in institutional core facilities and commercial laboratories, and through for-profit ventures, facilitating their increasing use in EHS and precision environmental health research.

#### Exposomics

The “exposome” represents a framework to study environmental drivers of health and disease^64^. The ultimate goal of exposomic science is to accurately define the totality of an individual’s chemical and non-chemical exposures (e.g., toxicants, diet, physical activity, and psychosocial stressors) over the lifecourse, including prenatally^64^. Rapid developments in analytical chemistry and other technologies provide highly sensitive, specific information on a wide range of external and exogenous exposures. For example, newer mass spectrometry technology enables untargeted screening of hundreds of chemicals at once in biological samples^65^. Also, the increasing abundance of environmental geospatial data, including air pollution, noise, and the natural, built, and social environments, allows for comprehensive characterization of external exposome factors^66^. However, the field continues to face numerous challenges in operationalizing the exposome, as the ideal goal to measure all exposures continuously throughout someone’s lifetime is still elusive.

#### Data science approaches

Advancing risk stratification and disease prevention using genomic, epigenomic, exposomic, proteomic, and metabolomic data will require data science approaches that integrate large datasets across multi-omic platforms. In this regard, artificial intelligence, which includes machine learning (ML) and natural language processing, can identify patterns in complex multidimensional datasets that include measurements of environmental exposures, and then apply that knowledge to identify individuals or populations most at-risk for adverse outcomes. ML is particularly well suited to unlocking knowledge and detailed information from semi-structured or unstructured data for precision environmental health, because time-dependent and high-throughput genome-scaled data types, such as epigenomic, transcriptomic, proteomic, or metabolomic data, are similar in structure. Some approaches are completely data-driven, only making the general assumption that the data will identify subgroups of individuals with characteristics of interest. For instance, ML approaches can help to differentiate cases and controls or to identify individuals with higher exposures and/or at increased risk of developing a certain disease. Hence, ML may reveal patterns that can inform personalized approaches that might not be discoverable using traditional statistical techniques^67^.

Because precision environmental health adds another dimension—the environment—to already high-dimensional data, robust data science approaches become even more critical to tease out the impacts of environmental exposures and develop approaches for risk stratification. As environmental exposures can affect biological processes at many different levels that contribute to disease, integrating multi-omic inputs in precision environmental health research can yield more comprehensive and accurate insights into how exposures impact health, for example, by moving beyond phenotype to endotype^68,69^ (Box 3). Approaches that allow for modeling the interactions among different “omic” data, such as the “interactome”, which uses a more multi-axial systems approach to identify the relevant networks that underlie environmental-driven diseases^70^, may provide additional advantages. Incorporating mechanistic understandings of how exposures contribute to disease, their associated molecular responses (endotypes), and “omic-based” determinants of disease risk, will enable the identification of target populations and individuals who will most benefit from targeted interventions—enabling a precision approach to disease prevention.

Box 3 Moving beyond phenotype to endotype Disease phenotypes are identifiable using a common rubric of key attributes such as clinical parameters and physiological characteristics. However, phenotype can be driven by different molecular mechanisms with disparate etiologies. Precision environmental health, by linking, for example, underlying individual risk factors and exposures contributing to disease pathogenesis with specific molecular (or other) endotypes, can provide an additional level of understanding of disease etiology, mechanisms of pathogenesis, and, potentially, new prevention and intervention strategies.

### Hallmarks of precision environmental health research

**Fig. 1:**
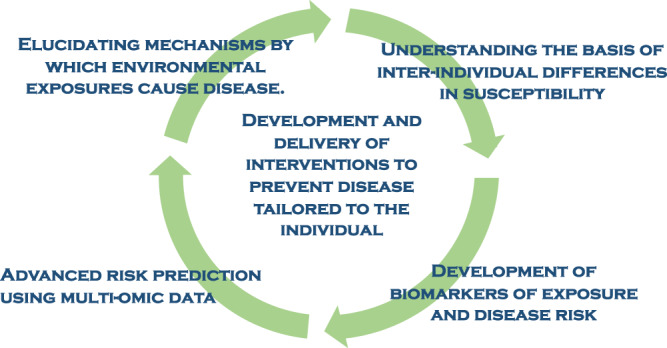
Hallmarks of precision environmental health. Precision Environmental Health seeks to prevent disease by understanding risk and tailoring interventions at the level of the individual. To reach this goal, precision environmental health research seeks to understand mechanisms by which environmental exposures cause disease and the basis for interindividual differences in susceptibility. This information will contribute to the attainment of this goal through the development of more precise biomarkers of exposure and risk and the development of advanced risk prediction models that incorporate “omic” data.

Precision environmental health has demonstrated its utility for the development and delivery of interventions and/or prevention of disease tailored to the individual in four key areas (Fig. 1).

#### Elucidating the complex mechanisms by which environmental exposures cause disease

Cells respond in diverse ways to environmental exposures^12^. Some of these responses, such as increased expression of metabolic genes to rapidly metabolize chemicals, directly reduce the impact of external exposures^5,21–23^. However, these adaptive responses also may cause adverse effects^12^. For instance, our body responds to a peak of air pollution exposure by mounting inflammatory responses locally in the lung and systemically to help clear air pollutants^71^. However, high repeated exposure and chronic exposures that induce a sustained inflammatory response can cause increased oxidative damage, a contributing factor to multiple diseases, including chronic lung disease, atherosclerosis and cardiovascular disease, accelerated cognitive aging, and dementia^3,72–74^. These responses are driven by a cascade of molecular, biochemical, and cellular changes that can be captured by various ‘omics approaches^12^. For instance, genetic polymorphisms may determine the robustness of an individual’s inflammatory response to air pollution, and how inflammatory genes are activated in response to air pollution can be influenced by an individual’s epigenome, which in turn controls changes in the transcriptome and the production of specific inflammatory proteins^75^. This single example illustrates how knowledge gained from genomic, epigenomic, transcriptomic, and proteomic data can help characterize responses to environmental exposures.

Other examples where the application of “omics” technologies are providing new insights into how environmental exposures contribute to the human disease include ExE interactions. Reprogramming of the epigenome by environmental exposure can persist long after the actual environmental exposure, sometimes even across multiple generations, and result in changes in the transcriptome, proteome, and metabolome^76,77^ that can drive adverse health outcomes. Therefore, while a variety of omics are influenced by the environment and mediate disease^78^, ExE interactions hold special promise for understanding how past exposures, especially those occurring early in life, influence the risk of disease later in adult life.

Studies in vitro and model organisms can also contribute mechanistic information linking exposures and adverse effects. While clinical sciences typically rely on randomized controlled trials to define the effects of treatment, comparable exposure–outcome trials are usually not ethically feasible^79^. For example, if observational data link prenatal pesticide exposure to adolescent cognitive deficits, one cannot design an ethical trial to expose pregnant women to that pesticide to determine if the relationship is causal. An alternative is to perform human intervention trials to remove the exposure^80,81^, a converse approach that can still reveal relevant information about the exposure–response relationship. However, some exposures are ubiquitous, and derived from multiple, widespread sources, making them challenging to control through simple interventions. Yet without causal data, how can observational data provide sufficient evidence to limit an exposure?.

**Fig. 2:**
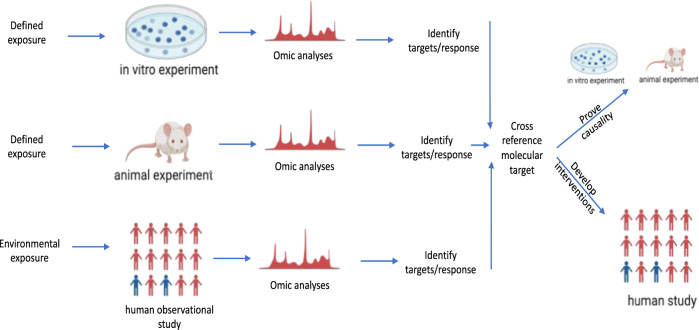
Translational framework of environmental epigenomics and precision environmental health. While human cohort studies can examine associations between environmental exposures and health outcomes, only surrogate tissues are available to assess molecular mechanisms involved in complex biological functions. Animal models can provide additional mechanistic insight by allowing for multi-tissue and multi-age comparisons. Animal models and in vitro cell culture studies can provide both mechanistic insights and a basis for moving beyond correlation to causality via reverse translation. Red people mean “unaffected”; blue people mean “susceptible”. Created with BioRender.com.

A possible path toward deriving mechanistic links and causality stems from sourcing correlation and mechanistic/causation data from separate complementary approaches rather than relying on just one type of study to supply all the answers. While human observational studies can provide rich data on the responses or targets of real-world environmental exposures, in vitro and in vivo experiments can provide similar insights from more defined, controlled exposures. Carefully controlled experiments in cell and animal models enable investigators to probe more deeply into the mechanisms by which exposures influence responses, which may not be feasible in population studies. By combining analyses from these parallel approaches, common molecular targets can be cross-referenced across population and laboratory studies, yielding a powerful strategy to mechanistically understand how real-world exposures influence health outcomes (Fig. 2).

### Predicting the risk caused by environmental exposures

#### Understanding the basis of interindividual differences in susceptibility

Current approaches in environmental health do not adequately incorporate emerging knowledge about how environmental exposures can alter individual disease risk^82^. For example, while the entire population of a large metropolitan area is exposed to the city’s air pollution, a relatively small proportion of individuals will develop diseases associated with air pollution exposure, such as cardiovascular disease, respiratory disease, or cancer^71,72^. Yet, we currently have limited means to ascertain individual risk or subpopulations disproportionately and adversely affected by air pollution exposure^83^. To lessen the burden of disease in response to this, and other adverse environmental exposures, we must identify those at greatest risk well before clinical effects are detectable to maximize preventive efforts.

#### Developing biomarkers of exposure and disease risk

Laboratory advances in exposome assessment have been powered by high-resolution mass spectrometry platforms that can measure many hundreds of exogenous chemicals, in addition to endogenous metabolites, in biological samples (e.g., blood, saliva, and urine)^84^. Operationalizing the exposome will be a major step toward fulfilling the promise of precision environmental health. While data continue to reveal the importance of the exposome, capturing exposures across the lifecourse is challenging^85^. Environmental exposures vary across time and tissues and, in most cases, must be sampled longitudinally (e.g., multiple times in a single day, across life stages) to accurately measure an individual’s exposure. Geospatial methods developed to characterize the external exposome factors are one approach to meet this challenge, and can model place-based characteristics of the environment over space and time to ideally estimate exposures such as air pollution, built environment, temperature, and noise, at any location and time. The growing capacity for personal monitors of environmental exposure (chemicals, pollen, extreme weather events, etc.) to collect real-time exposure data can provide opportunities for “personalized” decision-making (e.g., avoid an area with higher exposure, increase/decrease medication dose, contact healthcare providers, etc.). Other tools, such as the use of “digital twins”, already being adopted in precision medicine^86^, may prove equally useful in integrating the exposome into precision environmental health. Further, when capturing the exposome is not feasible, the evaluation of persistent epigenetic alterations may be possible as a proxy for exposures, and provide useful information for personalized decision-making. However, tools and applications are still limited and often constrained by the scarcity of data needed for evidence-based decision-making.

To this end, the Exposome-Explorer is a major advancement that offers the first database dedicated to biomarkers of exposure to environmental risk factors, including dietary factors, pollutants, and contaminants measured in population studies^87,88^. Exposome-Explorer has collected detailed information on the nature of biomarkers, their concentrations in various human biospecimens, the study population and the analytical techniques used for measurement, correlations with external exposure measurements, and data on biological reproducibility over time. This information can be used in precision environmental health research to compare the performance and field of application of various biomarkers and to identify the specific biomarkers or panels of biomarkers that are most useful for biomonitoring or disease etiology studies. Further, technologies that can measure and assess response to exposures at the molecular, protein, and metabolite levels are providing new opportunities to develop biomarkers of response to the same exposures. Combining knowledge of the mechanisms by which environmental exposures cause disease with exposure assessment and individual susceptibility factors can provide biomarkers of disease risk that can be applied to individuals and across populations.

Epigenetic biomarkers have also proven useful, as alterations caused by environmental exposures may persist long after the exposure ends, in some instances for a lifetime. Hence, epigenomic profiling can also provide both a record of past exposures and biomarkers for the risk of adverse health effects from environmental exposures at the level of the individual. One hurdle for incorporation of ExE studies in molecular epidemiology is the reliance of these studies on cells present in non-invasive biological samples, such as saliva, urine, stools, and meconium or biospecimens that can be obtained through minimally-invasive collection of blood, sputum, buccal and nasal epithelium from the respiratory tract, placenta and umbilical cord tissues, and breast milk^89–91^. Since the genome of cells present in specimens is identical to that of other cells across the human body, cells in those samples prove good surrogates for genetic studies. However, the epigenome is highly tissue- and even cell-type-specific^92,93^. Whether epigenetic alterations that occur in surrogate tissues in response to environmental exposures provide correlative information on the impact of these exposures on the epigenome of target tissues for the disease remains unclear.

To help assess the relationship between epigenomic alterations induced by the environment in target and surrogate tissues, the National Institute of Environmental Health Sciences (NIEHS) recently launched the TaRGET II: Environmental Epigenomic Analysis in Tissue Surrogates Consortium^94^ (Box 4). TaRGET II explores whether correlative epigenetic signatures induced by environmental exposures occur in both surrogate and target tissues using mouse models. Ultimately, this consortium intends to provide epigenomic data to help design and interpret epidemiology studies following inaccessible target tissues with available surrogate tissues. The success of these TaRGET studies will be key to developing future practical epigenetic applications for precision environmental health.

Epigenomic alterations in response to the environment not only have the potential to serve as reporters of past environmental exposures, but, as the epigenome is modifiable, opens the possibility of targeted epigenetic interventions. Recent developments in techniques that allow selective manipulation of a cell’s epigenome^95^ provide opportunities to use epigenetic editing to identify causal linkages for ExE interactions that drive disease and open possibilities for designing epigenetic interventions to prevent and treat environmental disease. However, challenges remain—existing methods for epigenetic editing can be globally indiscriminate and have significant off-target effects, or in the case of exogenous DNA, can be limited by the size of DNA recognition motifs^26,96^. The use of Piwi-interacting RNA (piRNA) begins to address these challenges^97,98^. piRNA is an endogenous mechanism for silencing genes and transposons at the transcriptional level, and can act permanently and acutely, unlike silencing using miRNA/siRNA, which act at the translational level and require repeated treatment.

#### Advanced risk prediction using multi-omic data

Precision environmental health seeks to provide a more accurate and individualized prediction of disease risk^99^. Combining knowledge of the environmental “riskscape”—which captures environmental exposures, the built environment, and demographic risk factors such as sex, race/ethnicity, and socioeconomic status—with knowledge of genetic, epigenetic, and lifestyle determinants of disease holds promise to more precisely assess individual risk and identify the most vulnerable individuals in a population, for example, to understand maternal and child health disparities^100^. Environmental exposures may have moderate to weak effects, and do not act in isolation to cause disease. Rather most exposures occur as mixtures, and, as discussed above, interact with multiple substrates and have “omic” effects on multiple substrates. This in contrast to single-gene disease-causing genetic mutations, which have strong and easy-to-identify effects^101^. However, despite these complexities, because environmental exposures influence everyone^102^, there is massive potential for precision environmental health to advance risk prediction and prevention strategies focused on environmental contributors to disease.

Population genetics has shown that disease risk is seldom determined by defects in only one or two genes. Rather, most common diseases involve multiple low-risk alleles and/or defects in genes that perturb multiple pathways. In genetics, this knowledge prompted the development of polygenic scores to predict disease risk, which combines multiple genetic variations into a single score that can help identify individuals at higher risk of disease. Analogous to polygenic contributions to disease, the adverse impacts of environmental exposures also could be quantified by creating data-driven “risk scores”. Such risk scores could be developed by integrating multi-‘omic inputs, including exposomics, genomics, epigenomics, metabolomics, proteomics, and metagenomic data, to reveal mechanistic information, contribute to the understanding of individual susceptibility to exposures, and inform risk prediction. Stratification based on such risk scores could then be used to identify those who will most benefit from targeted interventions to mitigate their exposures or ameliorate their adverse health effects—enabling a precision approach to disease prevention.

Together, these four hallmarks form the cornerstones for precision environmental health research. However, while research in these areas continues to advance, their application to public health practice is still constrained by the need for further information about their practical utility as well as challenges to large-scale implementation, as discussed below.

#### Precision environmental health and disease prevention

**Fig. 3:**
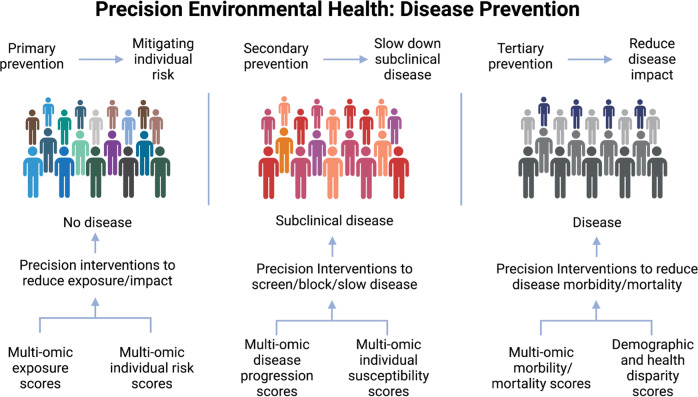
Precision environmental health applications to primary, secondary, and tertiary prevention. Precision environmental health research can contribute to primary prevention (left) by mitigating individual risk, secondary prevention (middle) by slowing the progression of subclinical disease, and tertiary prevention by reducing disease impact (right). Colors are used to indicate diversity within exposed populations. Created with BioRender.com.

Here we discuss opportunities for precision environmental health in disease prevention and outstanding challenges to bringing these approaches to fruition. Precision environmental health can provide a targeted approach to prevent disease by focusing exposure mitigation, interventions, and treatments at the level of the individual, and to those most vulnerable. Such strategies can be incorporated into primary, secondary, and tertiary prevention^103^ (Fig. 3).

#### Primary prevention

It refers to preventing the initial occurrence of disease, including limiting exposures to reduce the overall disease burden. Rather than an untargeted population-agnostic approach, precision environmental health approaches could discern who is at greatest risk of disease, for example, by establishing biomarkers that inform how specific exposures (or mixtures of exposures), will impact an individual based on their genome, epigenome, and microbiome.

**Fig. 4:**
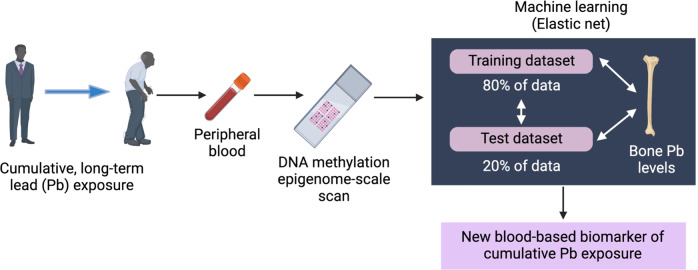
Use of epigenetic data and machine learning to develop a new blood-based biomarker of cumulative lead (Pb) exposure in the Boston area Veteran Affairs Normative Aging Study^1^. Epigenome-scale blood methylation data were used to train and test against benchmark bone-Pb data, which reflect long-term Pb exposure and were available in the study. This type of epigenetic biomarker holds great promise to capture and accurately assess long-term, cumulative exposures to environmental and lifestyle factors. Created with BioRender.com.

An example of a potential precision environmental health tool for primary prevention comes from ref. ^104^, who analyzed data from the Normative Aging Study to identify DNA methylation-based biomarkers that discern individuals with high levels of past lead exposure (Fig. 4). DNA methylation has practical advantages as an epigenetic biomarker as it can be measured on biospecimens obtained with a variety of collection methods (e.g., standard blood draw, dried blood spot, saliva kept at room temperature, etc.) and can be applied to archival samples using standard storage methods. In identifying differentially methylated regions associated with lead levels in patella and tibia bones, the authors were able to assess cumulative lead exposure (the elemental half-life of lead extends beyond a decade in bone). Correlating these lead measurements with methylation patterns measured in blood identified a signature that not only served as a surrogate measure of lead levels in bone, but also cumulative lead exposure across time. This signature could serve as a simple biomarker—much easier than obtaining measures of bone lead, which requires highly specialized equipment—to identify individuals for targeted interventions to ameliorate the long-term health effects of lead exposure, which include adverse cardiovascular, neuro-cognitive, and renal system effects.

Such surrogate biomarkers hold great promise for high-throughput screening of potentially at-risk populations and for guiding intervention efforts to those most susceptible; however, more information is needed about the applicability of these biomarkers to other exposures and populations, and the cost-effectiveness of such screening when applied across the population. While DNA methylation is easily assessed from a drop of blood, most DNA methylation platforms are designed for research laboratories and do not yet have the scalability and cost of typical clinical tests. This illustrates that while precision environmental health approaches can have great utility, research, and technological advances are still needed to support their broad adoption.

#### Secondary prevention

It offers opportunities to mitigate exacerbating effects of environmental exposures once the disease is initiated but still preclinical, by promoting early disease detection and providing targets to impede the environmental promotion of disease progression^105^. A precision environmental health framework could also guide screening efforts to target resources to those with disproportionately higher risk based on their environmental exposures. An example of the applicability of precision environmental health to secondary prevention comes from the Trial to Assess Chelation Therapy 2 (TACT2) study^106^, an ongoing clinical trial examining the outcomes of EDTA chelation therapy combined with oral vitamins and minerals to treat patients with diabetes who have experienced a previous cardiac event. The initial study, TACT1, found a reduction in recurrent cardiac events following a myocardial infarction when EDTA chelation therapy to remove lead and cadmium was combined with oral vitamins, with a particularly profound effect in a subset of the cohort who also had diabetes^107,108^. TACT2 is testing replication of these findings in a larger, diabetes-focused cohort and could lead to blood lead and urine cadmium levels being developed as biomarkers to screen cardiac patients who may benefit from EDTA chelation therapy.

#### Tertiary prevention

It aims to reduce disease symptoms and patient morbidity/mortality to improve an individual’s quality of life even in the context of established clinical disease^109^. Precision environmental health in the setting of tertiary prevention could, for example, help protect patients most susceptible to environmental triggers of morbidity/mortality. Exposure to PM_2.5_ (i.e., fine air pollution particles with aerodynamic diameter ≤2.5 μm) exemplifies this point—while PM_2.5_ exposure has negative health effects across the population, those effects become particularly dangerous in individuals with asthma, whose airways are inflamed, constricted, and susceptible to insult^83^. Because asthma is an umbrella term comprising various subgroups that require different therapies and management, precision environmental health can help identify asthmatics who may have worse exacerbation from PM_2.5_ exposures and might benefit most from interventions. These individuals, for example, might benefit most from avoiding areas with higher ambient exposures or reducing indoor levels in home, work, and school environments^110^.

Box 4 Toxicant exposures and responses by genomic and epigenomic regulators of transcription (TaRGET) consortium^26^ Launched by NIEHS, TaRGET I explored how adverse environmental exposures impact the epigenome. TaRGET II established a multi-institution consortium to validate the robustness and feasibility of using surrogate tissues (e.g., peripheral blood lymphocytes) to detect epigenetic reprogramming by early-life exposures in mouse models. The third phase, TaRGET III, will support the translation of epigenomic data from the mouse- and cell-based studies to human population-based studies with available epigenomic data. The fourth phase, TaRGET IV, will support integrated analyses in population-based studies, using several genomic and epigenomic databases to develop more comprehensive analyses.

### Conclusion

Precision environmental health holds great promise for enhancing our understanding of how the environment influences human health. Integrating environmental exposures across the lifecourse with large “omic” datasets using data science approaches, a precision environmental health framework has transformative potential for the development of precise and effective disease prevention. However, bringing precision environmental health into the realm of clinical and public health applications will require more action. Clearly, to realize the promise of precision environmental health will require training a workforce with the skills needed to work at the interface of the environment, the “omic” sciences such as genomics and epigenomics, and data science. In addition, the development and application of a precision environmental health framework will require additional ‘omic inputs from population, model organism, and in vitro systems, and technological refinements to translate knowledge and techniques from the laboratory to health practice. Most important, success will depend not only on scientific and technological advances but also on society’s capacity to incorporate advances in precision environmental health into public health strategies and individual health decision-making.
